# Chinese cross-cultural adaptation and validation of the Oxford shoulder score

**DOI:** 10.1186/s12955-015-0383-5

**Published:** 2015-12-03

**Authors:** Ximing Xu, Fei Wang, Xiaolin Wang, Xianzhao Wei, Zimin Wang

**Affiliations:** Orthopaedic Department of Changhai Hospital, Second Military Medical University, No 168, Changhai Road, Shanghai, 200433 People’s Republic of China; Department of Anesthesiology, Changhai Hospital, Second Military Medical University, Shanghai, 200433 People’s Republic of China

**Keywords:** Oxford shoulder score, Shoulder, Validation, Quality of life

## Abstract

**Background:**

The Oxford Shoulder Score (OSS) is a reliable and valid construct measuring non-specific shoulder pain, which are widely used to evaluate shoulder related quality of life. This study was to cross-culturally adapt and psychometrically validate a simplified Chinese version of the OSS (SC-OSS).

**Methods:**

Cross-cultural adaptation was performed according to the international recognized guidelines. Consecutive patients with nonspecific shoulder pain were recruited to test the psychometric properties of SC-OSS. Item response trend and item-total correlation were evaluated to measure homogeneity. Principal component analysis (PCA) was used to evaluate the factorial structure. Cronbach's α and intra-class correlations were used to determine the reliability. Construct validity was analyzed by evaluating the correlations between SC-OSS and the Constant-Murley shoulder outcome score (CMSOS), the short form (36) health survey (SF-36) containing eight domains, and pain visual analogue scale (VAS).

**Results:**

Overall, 121 patients were recruited. Each of the 12 items was properly responded and correlated with the total items. PCA extracted one factor for SC-OSS. SC-OSS had excellent reliability, with a Cronbach's α of 0.92 and intra-class correlation coefficient of 0.97 (95 % CI: 0.94-0.98). SC-OSS had a high correlation with CMSOS, physical functioning (PF) and bodily pain (BP) domains of SF-36 and VAS (*r* = -0.70, -0.65, -0.53, and -0.66, respectively). SC-OSS moderately correlated with role-physical (RP), social functioning (SF), general health perception (GH) and vitality (VT) (*r* = -0.45, -0.42, -0.39 and -0.36, respectively), but had a low correlation with role-emotional (RE) and mental health (MH) domains of SF-36 (*r* = -0.28 and -0.23, respectively).

**Conclusions:**

SC-OSS demonstrated excellent acceptability, internal consistency, reliability and construct validity, which can be recommended for application in mainland China.

## Background

Shoulder pain is a common disabling orthopedic condition experienced by many patients worldwide, just following low back pain and neck pain as the third most common disease in orthopedic visits [36]. The lifetime prevalence of shoulder pain in the general population is 6.7-66.7 % [22]. Shoulder pain is also a major problem in China. One previous study reported a 38.8 % one-year prevalence rate in the working population [43]. The most common causes of persistent shoulder pain are rotator cuff disorders, adhesive capsulitis, and glenohumeral osteoarthritis [25]. In addition, shoulder pain greatly affects arm function and leads to disability, which remains an enormous burden for the society [24, 25].

Recently, more and more healthcare providers are concerned about the quality of life of patients with shoulder pain [20, 37]. Interventions that better alleviate symptoms and improve shoulder functions are selected based on patients' reports [6, 37]. Therefore, measurement tools for patient-reported outcomes are essential and critical in the management of shoulder pain. Although there have been several validated self-reported questionnaires for low back pain and neck pain in China [39–42], scarce constructs are available for Chinese physicians to evaluate the status of shoulder pain and the effectiveness of interventions. To evaluate the quality of life in patients with shoulder pain, several constructs have been developed such as CMSOS [8], disabilities of the arm, shoulder and hand (DASH), simple shoulder test (SST), and shoulder pain and disability index (SPADI) [1]. Each has its advantages in measuring pain perception and functional disability. Currently, only CMSOS is available for Chinese patients [16, 23]. However, CMSOS can be affected by surgeon bias [30]. As a result, new patients-based constructs for patients with shoulder pain are needed. Therefore, it is imperative to develop a valid and easy administrative construct specific for Chinese patients with shoulder pain.

Cross-cultural adaptation of an existing measure may render the adapted measure as an objective uniform criterion for international or multi-center clinical trials [3, 17]. Due to differences in idioms and traditions in daily life, translation of a questionnaire directly from another language is inadequate for application of the questionnaire in a new culture. Therefore, cultural adaptation of the tool is vital, and psychometric properties must be tested [3, 17].

The Oxford Shoulder Score (OSS), first described by Dawson et al. in 1996, is a shoulder pain specific questionnaire for the evaluation of pain perception and daily function in patients suffering from shoulder pain, with excellent internal consistency, reliability and validity [10]. It has been widely accepted for its simplicity and easy administration for doctors and patients, and has been applied in several clinical conditions such as shoulder surgery [10], rotator cuff injury [34], and frozen shoulder [7]. Currently, it has been translated and validated in Dutch [4], German [19], Danish [15], Korean [28], Turkish [35], Norwegian [12] and Italian [26]. However, no valid simplified Chinese version is available.

Therefore, the aim of this study was to cross-culturally adapt and psychometrically evaluate the simplified Chinese version of OSS (SC-OSS) in patients with shoulder pain in mainland China.

## Methods

### Translation and cross-cultural adaptation

The English version of OSS was translated and cross-culturally adapted according to international guidelines for the process of cross-cultural adaptation of self-report measures [10, 17]. Owing to the acquirement of much experience in validation of many chronic pain related questionnaires by our group [40, 41], the employed process was just similar as the translation and back-translation method. Then, a pilot test of the pre-final version of SC-OSS was carried out in 30 patients with shoulder pain. Each patient completed SC-OSS and was subsequently interviewed about any difficulties in completing the questionnaire or understanding the purpose and meaning of each question. The expert committee, consisting of four forward and backward translators, two orthopedic surgeons, one rehabilitation physician, one physical therapist, one language expert and two patients with shoulder pain, reviewed the findings and developed the final SC-OSS (Appendix), which was subjected to further psychometric testing.

### Participants

Ethnical Han Chinese outpatients over 18 years of age with a confirmed diagnosis of non-specific shoulder pain for over 4 weeks due to rotator cuff pathology or inflammatory arthritis were eligible for recruitment in the Orthopedic Department at the Changhai Hospital between November 2013 and May 2014. Approximately 50 % of the eligible patients were randomly selected to take “test and retest” with an interval of 3 to 5 days. Patients who had shoulder pain with specified causes, such as shoulder instability, acute traumatic shoulder injury, tuberculosis or tumor, and who were unwilling to complete the questionnaires were excluded from the study. Demographic and clinical data and medical history were obtained, and a physical examination was performed at admission. Radiological images such as X-ray, computerized tomography, or magnetic resonance imaging were performed if they were required to evaluate the disease or confirm the diagnosis. The study was approved by the Human Research Ethics Committee of the Changhai Hospital, and written informed consent was obtained from every participant.

## Instruments

### OSS

OSS contains 12 items, each of which scores from 0 (worst) to 4 (best), according to a modified version [10], with the total score ranging from 0 (worst) to 48 (best). In the original version, each question was scored from 1 to 5, with 1 representing the best outcomes [10]. Unintuitive findings reported by surgeons using the original version led to the modified scoring system [11].

### CMSOS

CMSOS is widely used for assessing the outcomes of the treatment of shoulder disorders, especially with surgeries, including pain perception scoring, functional assessment, range of motion and strength measures [9]. CMSOS consists of four variables with two objective (pain and activities of daily life) and two subjective domains (active range of motion and shoulder strength). It has been trans-culturally adapted into Chinese with excellent reliability and validity [23].

### SF-36 health survey

SF-36 is widely applied to assess the health status of patients [38]. It contains eight domains, including physical functioning (PF), role limitations due to physical health (role-physical, or RP), bodily pain (BP), general health perception (GH), social functioning (SF), role limitations due to emotional problems (role-emotional or RE), vitality (VT), and mental health (MH). A higher score indicates a healthier status and less function loss. SF-36 has also been translated and culturally adapted into Chinese [21].

### Pain VAS

Pain VAS allows patients to rate their pain intensity on a 100 mm line anchored with two endpoints labeled 0 (No Pain) and 100 (Worst Possible Pain). It is highly accepted and widely used as a psychometric tool for its simplicity and good reliability, validity, and responsiveness [2].

### Psychometric testing

#### Response trend

The scores obtained for each of the items among the patients should be normally distributed, such that the mean score obtained on an item is close to the center of the possible response range of scores available for that item. Items with a mean score near the extreme of a possible range have low variance, whereas items that vary over a narrow range have poor correlations with other items [14].

Therefore, the standardized values of skewness (Z-skewness) were computed for each item. Any items with a skewness value within 1.96 were defined to have a response trend of normal distribution [14].

#### Ceiling and floor effects

Ceiling and floor effects were also analyzed, with such effects being present if over 15 % of participants achieved the highest or lowest score, respectively.

#### Homogeneity

A measure should be homogeneous; items should assess different facets of the same construct and correlate moderately with each other, and more importantly, each item should correlate with the total items. Therefore, internal consistency and item-total correlations were determined as functions of homogeneity. Internal consistency was calculated as Cronbach’s α, which was considered acceptable if the value was over 0.70 [27]. An item-total correlation coefficient was determined by Pearson’s correlation analysis. An item that had an item-total correlation coefficient less than 0.20 was eliminated [32].

#### Analysis for the factorial structure

The structure of SC-OSS was explored. Principal Component Analysis (PCA) with both varimax and oblimin rotations were adapted. The Kaiser-Meyer-Olkin measure and Bartlett's test of sphericity were conducted. The factorial structure was determined by the scree plot of the eigenvalues against the component numbers.

#### Test-retest reliability

To assess the reproducibility of SC-OSS, the intra-class correlation coefficient (ICC) was tested between the scores obtained at the first test and the second test. An ICC value was interpreted as good (>0.60) and excellent (> 0.80) reliability [31]. Also, a Bland-Altman plot was carried out to assess within subject variation and limits of agreement [5].

#### Construct validity

Construct validity was analyzed by Pearson’s correlation coefficients for the correlations between SC-OSS and other shoulder pain related measures (i.e. CMSOS, SF-36 and VAS). Correlation was interpreted as poor, fair, moderate, very good and excellent when r = |0.00-0.20|, r = |0.21-0.40|, r = |0.41-0.60|, r = |0.61-0.80| and r = |0.80-1.00|, respectively [13].

### Statistical analysis

It has been advised that at least 100 patients are necessary for internal consistency analysis and 50 for appropriate analyses of reliability and construct validity [33]. Therefore, over 100 patients were recruited in the study. Statistical analysis was performed using the Statistical Package for the Social Sciences (SPSS) version 18.0 (SPSS, Chicago, IL). Mean values are reported with standard deviation (SD), and ICC values are presented with 95 % confidence intervals (CIs). A *P* value of less than 0.05 was considered statistically significant for all analyses.

## Results

### Translation and cross-cultural adaptation

There were no major linguistic problems in the translation. Some discrepancies were encountered due to cultural differences. Specifically, the third item “car” was replaced by “private automobile,” as car has many meanings in Chinese such as small cars, sport-utility vehicle or jeep. “Chopsticks and spoons” were substituted for the fourth item “knife and fork” because Chinese usually do not use a knife and fork. Some male participants reported that they did not do household work so the fifth item was amended as “if you are doing the household shopping, could you do it on your own”. In addition, “bowl” was used to replace the sixth item “tray” since most Chinese people commonly use a bowl to hold food.

### Characteristics of patients

Overall, 121 eligible patients were recruited in the study. Of the eligible patients, 55 were selected and participated in the test-retest. The mean completion time was 2.1 (±1.2) minutes. Nearly all of the participants filled out the questionnaire properly. Only 1 or 2 items were left blank in 18 participants with a responding rate was 95.9 % (1426/1452) for total items. The clinical and demographic characteristics of the patients are summarized in Table 1.

**Table 1:** Baseline demographic and clinical characteristics of patients with non-specific shoulder pain

Variables	Values
Gender	
Male/Female	51/70
Age (Mean ± SD, year)	51.2 ± 8.9
Pain duration (Months)	7.9 ± 5.6
Shoulder dominant Left/Right	57/64
Education	
Primary school	42
Middle school	23
High school	27
University/colleg5.e	19
Diagnosis	
Subacromial impingement	34
Rotator cuff syndrome	48
Tendinitis	10
Adhesive capsulitis	19

### Response trend and ceiling and floor effects

Z-skewness showed that none of the items were distributed outside 1.96. The item-total correlation analysis revealed that each item had a moderate to good item-total correlation, and none had a Pearson coefficient of less than 0.30. Detailed results are demonstrated in Table 2. No ceiling or floor effect was detected in the total or separate items.

**Table 2:** Respond trend, corrected item-total correlation and factor loading for each item of SC-OSS

Item	Z-skewness	Corrected item-total correlation (r)	Factor loading
1	0.13	0.62	0.69
2	0.99	0.69	0.75
3	0.89	0.67	0.73
4	1.19	0.70	0.76
5	1.20	0.63	0.69
6	1.14	0.69	0.75
7	1.15	0.56	0.63
8	0.51	0.71	0.77
9	0.98	0.77	0.82
10	0.35	0.65	0.71
11	0.19	0.70	0.76
12	1.00	0.71	0.76

### Factorial structure

**Fig. 1:**
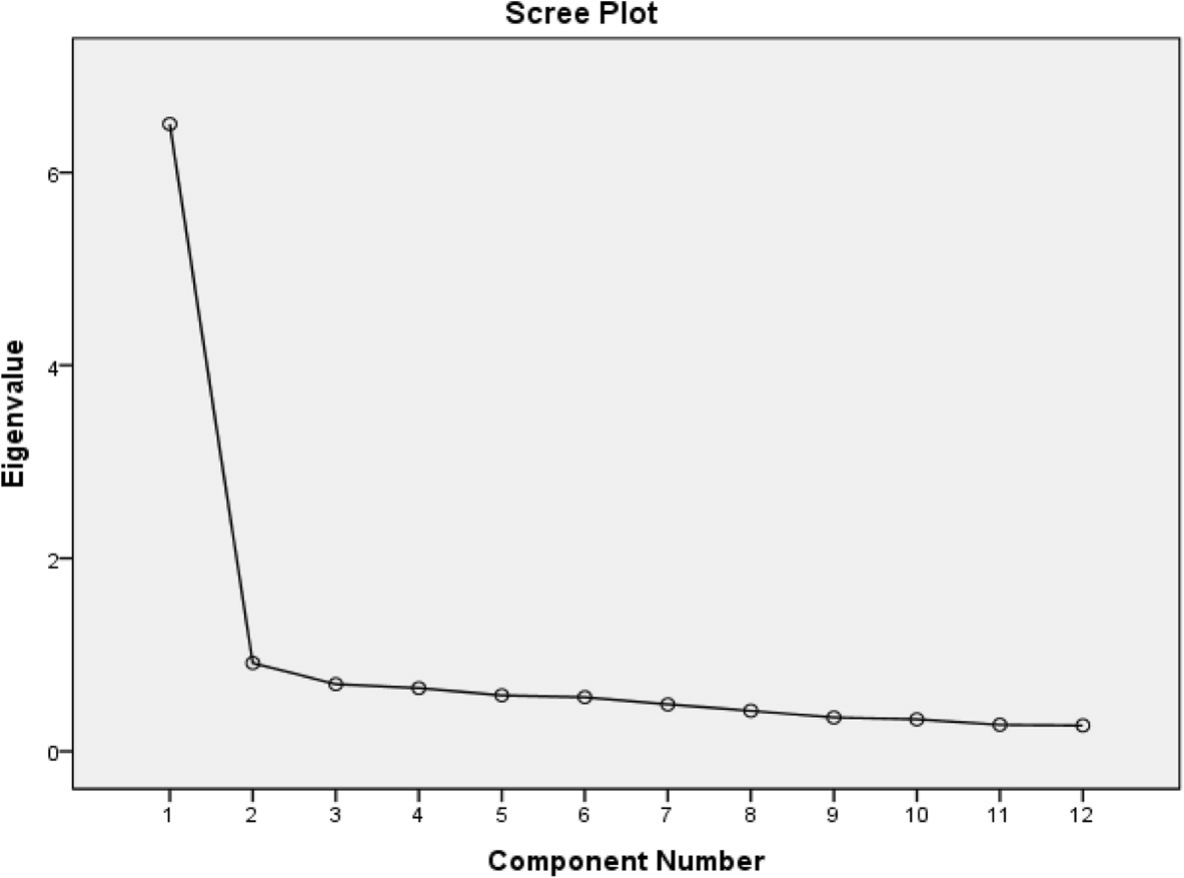
The scree plot of the eigenvalues against the component numbers for SC-OSS

The Bartlett’s Test of sphericity failed to show that the correlation matrix was suitable for analysis of the factorial structure with adjustment (*P* < 0.001). Both the eigenvalues and the scree plot (Fig. 1) suggested one latent factorial structure, which jointly accounted for 54.2 % of the total variance.

### Reliability

**Fig. 2:**
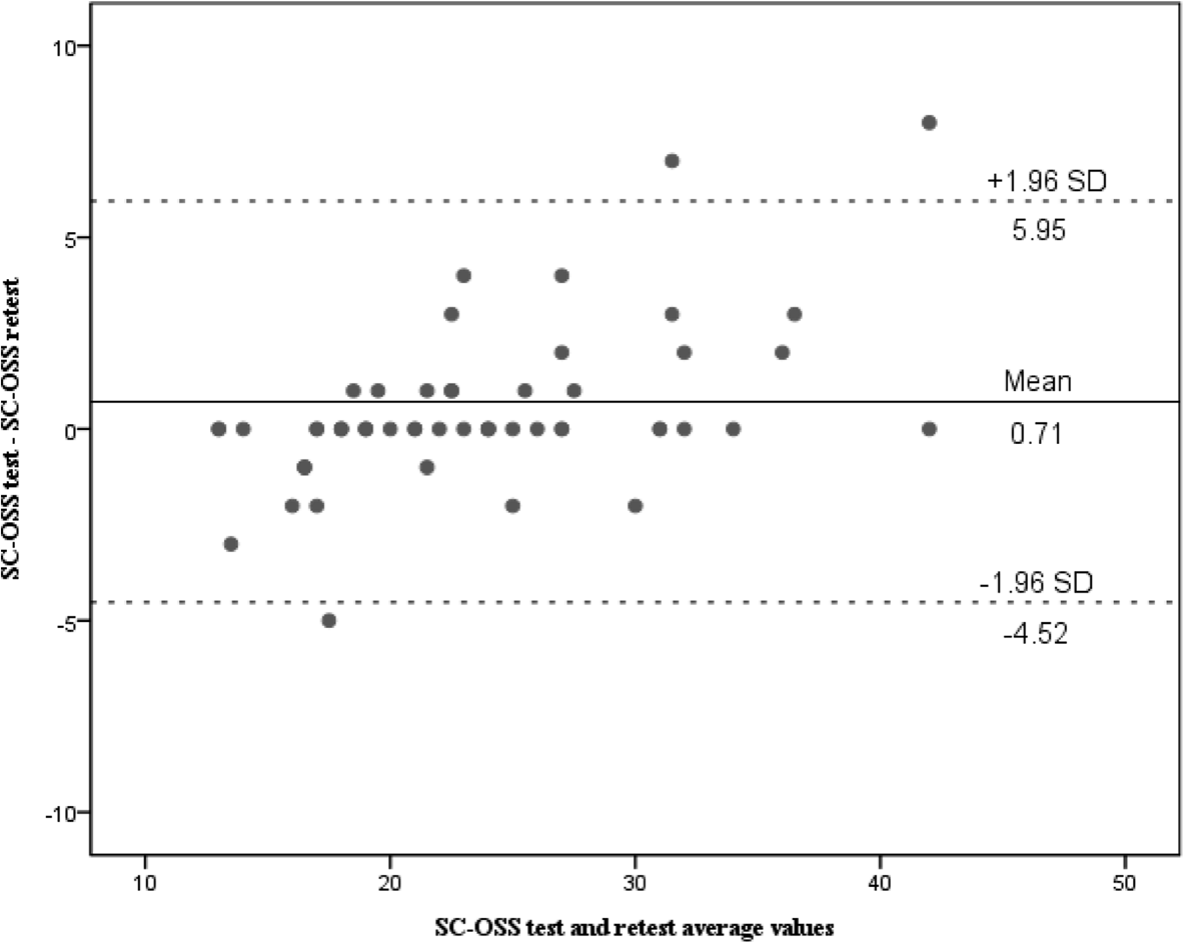
The Bland-Altman plot for test-retest agreement of SC-OSS. The differences between patients for SC-OSS from two test sessions were plotted against the mean of the two session total scores. The line indicates the 95 % (±1.96 standard deviation) limits of agreement

High internal consistency and test-retest reliability were confirmed, as the Cronbach’s α was 0.92 and the ICC was 0.97 (95 % CI: 0.94-0.98) for SC-OSS. The Bland-Altman plot demonstrated the differences between scores from the two test sessions for the individual patients and the overall means of the two sessions (Fig. 2). No systematic bias was found, indicating favorable test-retest agreement of SC-OSS.

### Construct validity

SC-OSS had correlated highly with CMSOS, VAS, and PF and BP domains of SF-36 (*r* = -0.66,-0.70, -0.65, and -0.53, respectively). SC-OSS correlated moderately with domains of SF-36 relating to function loss due to chronic pain, including RP, SF, GH and VT (*r* = -0.45, -0.42, -0.39 and -0.36, respectively). Finally, SC-OSS had low correlation with domains of SF-36 unrelated to shoulder pain, including RE and MH in SF-36 (*r* = -0.28 and -0.23) (Table 3).

**Table 3:** Comparison on reliability and construct validity among different versions of OSS

	Chinese (*n* = 121)	English (*n* = 111)	Dutch (*n* = 103)	German (*n* = 94)	Italian (*n* = 140)	Turkish (*n* = 84)	Norwegian (*n* = 74)	Danish (*n* = 102)	Korean (*n* = 105)
Reliability									
Cronbach’s α	0.92	0.89-0.92	0.92	0.98	0.95	0.92	0.87	0.93	0.91
ICC (95 % CI)	0.97 (0.94-0.98)	NA	0.98 (0.96-0.99)	NA	0.97	0.99 (0.93-0.99)	0.83 (0.73-0.90)	0.98	0.95 (0.91-0.98)
Construct validity									
VAS	0.70	NA	NA	NA	NA	NA	NA	NA	0.34- 0.45
CMSOS	0.66	0.74	0.64	0.60	0.73	NA	NA	NA	0.42- 0.68
SF-36									
PF	0.65	0.61	0.68	0.62	0.74	0.63	NA	NA	NA
RP	0.45	0.41	0.46	0.56	0.66	0.38	NA	NA	NA
BP	0.53	0.66	0.56	0.76	0.58	0.74	NA	NA	NA
VT	0.36	0.52	0.20	0.49	0.49	0.44	NA	NA	NA
SF	0.42	0.55	0.25	0.45	0.64	0.49	NA	NA	NA
RE	0.23	0.37	0.38	0.27	0.55	0.33	NA	NA	NA
MH	0.28	0.39	0.15	0.54	0.56	0.42	NA	NA	NA
GH	0.39	0.34	0.10	0.39	0.40	0.39	NA	NA	NA

*ICC* intra-class coefficient, *VAS* visual analogue scale, *CMSOS* Constant-Murley shoulder outcome score, *PF* physical functioning, *RP* role limitations due to physical health, *BP* bodily pain, *GH* general health perception, *SF* social functioning, *RE* role limitations due to emotional problems, *VT* vitality; and *MH* mental health, *NA* not available

## Discussion

In the present study, the English version of the OSS was successfully adapted and psychometrically validated into simplified Chinese. Statistical analysis revealed that all items of SC-OSS were well distributed and moderately correlated with each other, with excellent internal consistency, test-retest reliability and construct validity. Factor analysis demonstrated that SC-OSS was uniform and well-structured for shoulder pain in Chinese patients.

To evaluate the quality of life in patients with shoulder pain, several constructs have been developed such as CMSOS [8], OSS [10], disabilities of the arm, shoulder and hand (DASH), simple shoulder test (SST), and shoulder pain and disability index (SPADI) [1]. Each has its advantages in measuring pain perception and functional disability. Currently, only CMSOS is available for Chinese patients [16, 23]. However, CMSOS can be affected by surgeon bias [30]. Specifically, two components of CMSOS are evaluated by the doctor, which leads to major variations among clinical settings [30]. Also, the reliability of CMSOS has been challenged due to the lack of standardization in the assessment procedures [8, 30]. As a result, new patients-based constructs for patients with shoulder pain are needed. Of the other four constructs mentioned above, the OSS is the most cross-culturally adapted and validated and has been proven to be simple, acceptable and reliable in many different cultures and areas [4, 13, 16, 21, 27, 29, 36]. Hence, we decided to cross-culturally adapt the OSS, for Chinese patient with shoulder pain, as well as provide an international validated tool for multi-center research on quality of life.

In the present study, the acceptance of SC-OSS was high as all the items were well responded by Chinese patients with non-specific shoulder pain, with a responding rate of 95.9 %. High responding rates were also demonstrated in Italian [26], Turkish [35], German [19] and Korean [28] patients for their corresponding version. In the present study, the mean completion time was 2.1 min, similar to that reported for the Turkish and Korean versions. A longer completion time (3 min and 25 s) was reported for the German study, and a completion time was not reported in the Dutch and Italian studies. The short completion time and high responding rate indicates that SC-OSS is highly acceptable, and thus could be easily administered with little effort.

During the cross-cultural adaptation process some minor modifications had to be made due to cultural differences between Chinese and Western patients. Homogeneity analysis revealed that none of the items should be omitted. Item-total correlation demonstrated that each item made a contribution to the sum of the construct. Similar results were also found in English [10], Turkish [35], Korean [28] and German^190^ studies. The English study applied the ceiling or floor effect, which has a similar statistical meaning to the response trend, and also showed good homogeneity [8]. Homogeneity analyses were not reported in the Italian [26] and Dutch [4] studies. Therefore, SC-OSS is homogeneous in measuring shoulder pain in a Chinese cultural background.

OSS was designed to assess the function of shoulder movement. The total items could be divided into two subgroups: pain related items and interferences of shoulder function related items. Yet, one factor structure was obtained in SC-OSS. No description of facture analysis were reported in other versions of OSS, including Dutch [4], German,19 Danish [15], Korean [28], Turkish [35], Norwegian [12] and Italian versions [26]. Therefore, the factor structure of OSS needs more investigation in different cultural settings.

SC-OSS had good internal consistency and reproducibility, indicating excellent reliability. The Cronbach’s α was 0.92, almost the same as that reported in the English and other language versions, indicating that OSS remains stable in different cultures. In addition, the ICC was 0.97, which is also consistent with that reported in most studies, suggesting that the OSS remains stable over time.

The construct validity of SC-OSS was tested against CMSOS, VAS and SF-36, as these constructs are commonly used in China. As expected, SC-OSS highly correlated with CMSOS, similar to the English, Dutch, German, Italian and Korean studies. However, regarding the correlation with VAS, the Korean study [28] verified a low to moderate correlation (*r* = 0.34), whereas our study observed a high correlation (*r* = 0.70). This discrepancy is probably due to the difference in patient demographic characteristics (i.e. a female dominant sample with younger age in our study versus a male dominant sample with older age in the Korean study). However, since no other studies reported a correlation between OSS and VAS, this assumption should be further tested and other factors contributing to the discrepancy should also be explored. SF-36 has been applied worldwide for evaluating patient quality of life, especially in chronic pain patients [18]. It has been used in most studies evaluating the construct validity of the OSS. In the present study, SC-OSS highly correlated with PF and BP, moderately correlated with RP, SF, GH and VT, and had a low correlation with RE and MH. Since the OSS focused on shoulder pain perception and functional disability, it is reasonable that SC-OSS has correlated highly with PF and BP, but had a low correlation with RE and MH. This phenomenon has also been demonstrated in the original English OSS version [10], indicating that the OSS has excellent construct validity across different cultures. However, it should be noted that the English version [10], Italian [26], Dutch [4] and German [19] versions adapted a 1 to 5 rating scale, whereas the Turkish [35] and present study adapted a revised scoring 0-4 rating scale. The different scoring may have led to slight discrepancies in evaluating the correlations between the OSS and other instruments.

There are some limitations in this study. First, the patients in this study are from a single center, which may not fully represent the whole country. Multi-center large sample study is favored. Second, responsiveness (i.e. sensitivity to change, reflects the ability to detect clinically significant changes) of SC-OSS was not determined, so as the German, Korean, Turkish and Italian studies.

## Conclusions

SC-OSS was successfully cross-culturally adapted and psychometrically validated in Chinese patients with non-specific shoulder pain. SC-OSS demonstrates high acceptance, excellent internal consistency, reliability, and solid construct validity. These findings provide Chinese surgeons and investigators with a tool to evaluate patients with non-specific shoulder pain.

## Abbreviations

OSS: Oxford shoulder score
SC-OSS: Simplified Chinese version of the OSS
PCA: Principal component analysis
CMSOS: Constant-Murley shoulder outcome score
SF-36: he short form (36) health survey
PF: Physical functioning
RP: Role-physical
BP: Bodily pain
GH: General health perception
SF: Social functioning
RE: Role-emotional
VT: Vitality
MH: Mental health
VAS: Visual analogue scale
ICC: Intra-class correlation coefficient
CIs: Confidence intervals
DASH: Disabilities of the arm, shoulder and han
SST: Simple shoulder test
SPADI: Shoulder pain and disability index
